# Java Web Start based software for automated quantitative nuclear analysis of prostate cancer and benign prostate hyperplasia

**DOI:** 10.1186/1475-925X-4-31

**Published:** 2005-05-11

**Authors:** Swaroop S Singh, Desok Kim, James L Mohler

**Affiliations:** 1 University of North Carolina Lineberger Comprehensive Cancer Center, University of North Carolina at Chapel Hill, Chapel Hill, USA; 2 School of Engineering, Information and Communications University, Daejeon, Korea; 3 Department of Pathology and Laboratory Medicine, University of North Carolina at Chapel Hill, Chapel Hill, USA; 4 Department of Urologic Oncology, Roswell Park Cancer Institute, Buffalo, USA; 5 Department of Urology, State University of New York at Buffalo, Buffalo, USA

## Abstract

**Background:**

Androgen acts via androgen receptor (AR) and accurate measurement of the levels of AR protein expression is critical for prostate research. The expression of AR in paired specimens of benign prostate and prostate cancer from 20 African and 20 Caucasian Americans was compared to demonstrate an application of this system.

**Methods:**

A set of 200 immunopositive and 200 immunonegative nuclei were collected from the images using a macro developed in Image Pro Plus. Linear Discriminant and Logistic Regression analyses were performed on the data to generate classification coefficients. Classification coefficients render the automated image analysis software independent of the type of immunostaining or image acquisition system used. The image analysis software performs local segmentation and uses nuclear shape and size to detect prostatic epithelial nuclei. AR expression is described by (a) percentage of immunopositive nuclei; (b) percentage of immunopositive nuclear area; and (c) intensity of AR expression among immunopositive nuclei or areas.

**Results:**

The percent positive nuclei and percent nuclear area were similar by race in both benign prostate hyperplasia and prostate cancer. In prostate cancer epithelial nuclei, African Americans exhibited 38% higher levels of AR immunostaining than Caucasian Americans (two sided Student's t-tests; P < 0.05). Intensity of AR immunostaining was similar between races in benign prostate.

**Conclusion:**

The differences measured in the intensity of AR expression in prostate cancer were consistent with previous studies. Classification coefficients are required due to non-standardized immunostaining and image collection methods across medical institutions and research laboratories and helps customize the software for the specimen under study. The availability of a free, automated system creates new opportunities for testing, evaluation and use of this image analysis system by many research groups who study nuclear protein expression.

## Background

Benign prostate hyperplasia affects most men and often causes lower urinary tract symptoms. Prostate cancer leads in the number of estimated new cases diagnosed each year and is the second leading cause of cancer specific mortality in American men [1]. The development of the prostate, benign enlargement of the prostate and prostatic carcinogenesis and progression require androgenic stimulation [2]. Since androgen acts via androgen receptor (AR), AR expression in prostatic cells may play a central role in prostate pathology. AR belongs to the steroid receptor superfamily, which includes estrogen, progesterone, corticosteroids, vitamin D, thyroid, and retinoic acid receptors [3]. A study using radiolabeled natural and synthetic AR ligands showed the presence of AR in normal prostate, benign prostate and prostate cancer [4]. High levels of AR are associated with increased proliferation, markers of aggressive disease and are predictive of decreased biochemical recurrence-free survival independently, confirming the role of AR in tumor growth and progression in hormonally naive prostate cancer [5].

AR protein expression was assessed first using visual scoring of immunostained sections of prostate tissue. A visual scoring technique developed by Miyamoto et al. [6] assessed intensity of AR immunostaining on a scale of 0 (none) to intense (3+) for each nucleus. However, recognition of malignant nuclei requires a skilled pathologist or a highly trained technician and visual assignment of immunopositivity is subjective, tedious and poorly reproducible. Mean optical density (MOD) measured using image analysis was found more accurate and reproducible for measuring AR expression but object identification remained difficult. Investigators used a light pen to encircle [7] or a sampling window [8] to select areas of malignant nuclei for optical density measurement. However, these interactive image analysis methods are tedious and user bias influences the measurements. Tilley et al. [9] used an automated color video image analysis system to measure MOD of each positively stained area in visually marked malignant tissue. MOD was calculated as the total integrated optical density divided by the area. Such measurements tend to be less accurate when compared to MOD measured from individual nuclei. In addition, MOD depends on variability of immunostaining intensity among tissue sections and tissue thickness [7-9].

Segmentation of prostatic epithelial nuclei from complex histological images and classification of nuclei as malignant or benign posed a significant challenge to accurately quantifying AR expression by image analysis. This barrier was overcome by combining segmentation algorithms and nuclear morphometry. Qualitative nuclear morphometry was used to characterize the aggressiveness of prostate cancer [10,11]. Kim et al [12] developed a semi-automated technique to identify individual prostatic epithelial nuclei and classify each nucleus as benign or malignant using nuclear shape descriptors. Segmentation of malignant prostatic nuclei allowed application of conventional image analysis algorithms to measure AR expression. A combination of CAS-200 image analysis system with Cell Sheet v2.0 and nuclear morphometric descriptors (NMD) has been used to develop quantitative nuclear grade (QNG), for making clinical, diagnostic and prognostic outcome predictions in prostate cancer [13].

Macros written in commercially available image analysis software such as Adobe Photoshop [14], Optimas [15] and Image Pro Plus [16] have also been used to perform semi-automated image analysis. User interaction is required for object (nuclei) selection, modification of object boundaries and/or selection of thresholds. Dedicated image analysis systems like CAS-200 [17], ACIS [18] and Autocyte [19] are employed in the determination of nuclear features from immunostained images. These systems use dedicated hardware and proprietary software and typically require manual interaction. Schnorrenberg et al. [20] developed an algorithm for automated analysis of breast cancer biopsies. The algorithm transforms the color image into bimodal distribution and identifies the presence and intensity of only the positively immunostained nuclei. Xu et al. [21] developed a software package for automated labeling of rat liver nuclei by integrating various commercial software using macros and Visual Basic. Loukas et al [22] used LabWindows libraries to develop an application for automated counting of nuclei in squamous cell carcinomas of the head and neck.

Our image analysis system was developed on networked DEC 5000 workstations, and used programs written in C++, Windows-based Statgraphics 4.1 and Optimas 6.0 [23-25]. The C++ programs incorporated graphics and image processing libraries (IGLOO) [26]. The image analysis programs were later ported to a PC compatible system on a Linux platform. However, images were collected using Windows-based systems that required image transfer across platforms. The current image analysis software was developed on a Java platform and incorporates all features of previous versions. The software eliminates the need for Statgraphics and Optimas and can be deployed on a variety of platforms with several versions of the software running at the same time without conflict [27]. The program is associated with a Web browser and can be downloaded freely from our website [28]. Once downloaded, Web connection is no longer required.

The image analysis software can be divided into four modules: a) detection of potential prostate nuclei; b) removal of artifacts; c) classification of prostate epithelial nuclei and d) measurement of AR expression. Human intervention is only required to create a set of classification parameters. These parameters are used to reduce the effect of local variations in slide preparation and image acquisition on nuclear measurements. The software identifies artifacts, distinguishes epithelial prostate nuclei from endothelial and stromal nuclei and inflammatory cells based on nuclear size and shape. Classification parameters are used to differentiate between immunopositive and immunonegative nuclei. AR expression is quantified by (a) percentage of immunopositive nuclei; (b) percentage of immunopositive nuclear area; and (c) intensity of AR expression among immunopositive nuclei or areas.

The development and operation of an automated image analysis system are described. A preliminary study comparing AR expression in paired specimens of benign prostate and prostate cancer from 20 African and 20 Caucasian Americans is presented to demonstrate an application of this system.

## Hardware and Software

### 1. Hardware specifications

The imaging system consists of a Leica DMRA2 microscope (Leica Microsystems Inc, Bannockburn, IL) with a Ludl motorized stage controller (Ludl Electronic Products Ltd, Hawthorne, NY), a Hamamatsu 3 Chip CCD camera with controller (Hamamatsu, Bridgewater, NJ) and a Flashpoint 3D image grabber card (Integral Technologies, Indianapolis, IN) in a Pentium IV based PC. The PC used is a Dell Optiplex GX240 (Dell Inc, Round Rock, Texas) with 1.8 GHz Pentium IV processor and 512 Mb RAM. Image Pro Plus 4.5 (Media Cybernetics Inc. Silver Spring, Maryland) software on Windows 2000 (Microsoft Corp, Redmond, Washington) platform was used for image acquisition and Linux based Dell Precision 530 and Windows based Dell Precision 340 were used for image analysis.

### 2. Software

Java Runtime Environment (Sun Microsystems, Santa Clara, CA) provides the platform to run the image analysis software. Once Java has been installed, the image analysis software can be launched from our web page [25]. Once it is cached on the local computer, Web connection is not required.

#### 2.1. Macro for obtaining RGB data

A macro written in Visual Basic (Microsoft Corp, Redmond, WA) is used to obtain area and RBG color information from selected regions of the image. The macro was developed in Image Pro Plus 4.5. The macro prompts the user at every step of the process. The user is prompted to open an image and select immunopositive and immunonegative nuclei. The macro guides the user to select individual nuclei using tools provided by Image Pro. The macro calculates the area (in pixels) and the mean RGB color values (0–255) of the pixels enclosed in the selected region. The data is automatically transferred to Microsoft Excel. A numerical tag is attached to each set of values from immunopositive and immunonegative nuclei.

#### 2.2. Overview of application software

The application program is divided into two modules.

##### A. Classification table

Data obtained from the macro (Section 2.2.1) is input to the program under 'Parameter Training'. The program checks the input data file format and displays it in a new window. The results of the analysis, 'Classification Table', are shown in a new window. Once the user is satisfied and accepts the results, a new set of classification parameters is created and stored.

##### B. Image analysis

**Figure 1:**
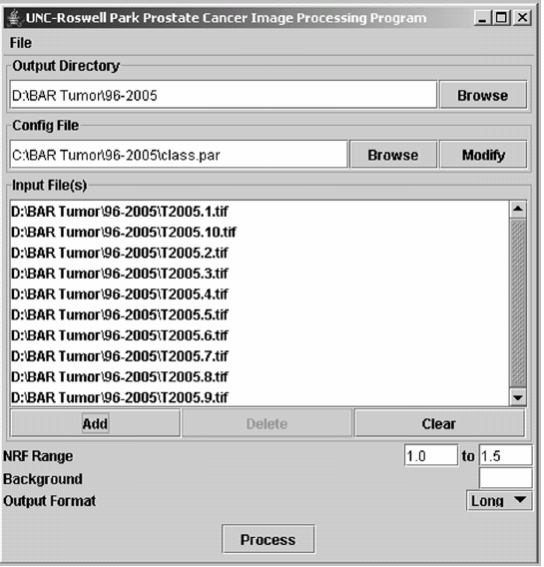
Graphical user interface (GUI) of the image analysis software.

If a classification parameter dataset already exists, it is loaded into the program by clicking on 'Browse' and locating the file. Parameters can also be entered manually by clicking on 'Modify' (under Config File). The location of the output directory is set by clicking on 'Browse' under Output Directory. Files are added and removed by clicking on 'Add' and 'Delete' buttons on Input Files frame. The output data files are stored in short (final results only) or long (results from individual nuclei) format with the same prefix as the input image file name. Images created during image analysis can be reviewed and stored by checking corresponding boxes under File in the main window. The graphical user interface (GUI) of the image analysis software is shown in figure 1.

## Methods

### 1. Study specimens

Prostate tissue specimens were obtained from archived, paraffin-embedded blocks of radical prostatectomy specimens. The expression of AR in paired specimens of benign prostate and prostate cancer from 20 African and 20 Caucasian Americans was compared to demonstrate an application of this system. Clinical data from these specimens was obtained from prospectively maintained clinical databases.

### 2. Immunohistochemistry

Immunohistochemistry allows for *in situ *protein localization and computer assisted image analysis of AR while preserving tissue architecture. Polyclonal or monoclonal antibodies target specific epitopes located within cellular structures to visualize epitopes of interest. Archival paraffin-embedded prostate specimens were cut into 6 *μ*m sections and placed on ProbeOn Plus™ microscope slides (Fisher Scientific, Pittsburg, PA). After deparaffination and rehydration through graded alcohols (100%, 95%, 70%), tissue sections were subjected to antigen retrieval in Reveal Citra buffer (Biocare Medical, Walnut Creek, CA) using a pressurized antigen-decloaking chamber for 2 minutes at 120°C and 21 PSI. The sections were cooled to room temperature and blocked for non-specific staining with 2% normal horse serum for 15 minutes at 37°C. Endogenous peroxidase was blocked using 3% hydrogen peroxide diluted in methanol and endogenous biotin was blocked using an Avidin Biotin kit (Vector, Burlingham, CA). Sections were incubated using a capillary gap method with monoclonal antihuman AR antibody F39.4.1 (Biogenex, San Ramon, CA) diluted in Primary Antibody Diluting Buffer (Biomeda Corp., Foster City, CA) at 1:500 for 1 hour at 37°C in a humidified heating block. Sections were incubated with biotinylated anti-mouse IgG (Vector) 1:200 for 30 minutes at 37°C. The signal was then amplified using avidin-biotin complex (ABC) Vector and visualized using 3,3'-diaminobenzidine (DAB) (Vector). Counterstaining was performed using hematoxylin (Fisher Scientific) for 15 seconds (diluted 1:3 in H_2_O). Slides were dehydrated through graded alcohol and mounted using Permount (Fisher Scientific). Benign and malignant tissues were immunostained in a single batch.

### 3. Image acquisition

The images were acquired using a 40:1 objective, N.A. 0.85 for a total magnification of 400×. Contrast and brightness were adjusted by manipulating the gain and exposure time of the camera. Illumination was adjusted to generate maximum contrast while avoiding over- and under-saturation of gray levels. A series of neutral filters were added to confirm the linearity of output in final optical settings. Temporal variation of light output was measured frequently and found insignificant (<0.2%). Images were sampled randomly throughout histological sections, but areas of necrosis, artifacts and edges were avoided. Each image was captured under the same reproducible conditions. White and Black balance of the camera was performed to ensure the optimal use of the dynamic range of the camera. Ten images were collected from each tissue specimen. Each image consisted of 640 × 480 pixels collected in 24-bit color mode (16.7 million colors) and was stored in an uncompressed tagged image format file (TIFF).

### 4. Creation of classification parameters

**Figure 2:**
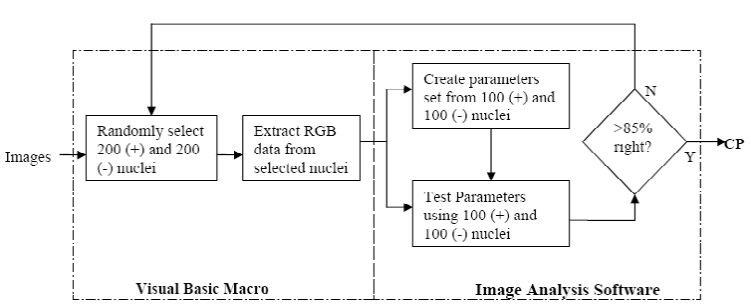
Steps involved in the creation of classification parameters. CP – Classification Parameters.

Commercial reagents used in the immunohistological staining process are not standardized; thus immunostaining patterns differ between various research labs. When combined with local variations in image acquisition, the resulting automated analysis may produce significant errors. Classification parameters are used to calibrate the nuclear analysis software with each new dataset, thus making the automated image analysis software independent of the type of immunostaining or imaging system used. Figure 2 shows the block diagram of the steps involved in the creation of classification parameters.

#### 4.1 RGB dataset

A minimum of 200 immunopositive and 200 immunonegative nuclei were randomly sampled from the acquired images. Red, Green and Blue information was extracted from the selected nuclei. A new column 'Class' was added to the dataset. Objects identified as immunopositive are class 1 and objects identified as immunonegative are class 2.

#### 4.2 Classification parameters

The dataset was divided into two non-overlapping sets; (a) training set and (b) test set. Each consists of at least 100 immunopositive nuclei and 100 immunonegative nuclei. Classification coefficients were computed from the training set using either of the following methods.

##### (a) Linear Discriminant Analysis

Let x = {x_hue_, x_saturation_, x_intensity_} denote individual data structure present and *x*_1 _= {x_11_, x_12_, x_13_..., x_1n1_}, *x*_2 _= {x_21_, x_22_, x_23_..., x_2n2_} represent class 1 and class 2 datasets from the training set with *μ*_1_, *μ*_2 _as their corresponding means [29].

The covariance of *x*_1 _and *x*_2 _is:





The pooled covariance is given by:

S_p _= (S_1 _+ S_2_) / (n_1 _+ n_2 _- 2)     (3)

where n_1_, n_2 _are the number of immunopositive and immunonegative nuclei in the training set, respectively.

The classification coefficients *λ *= {*λ*_1_, *λ*_2_, *λ*_3_} and constant C are computed as



C = *μ λ *    (5)

where S^-1 ^is the inverse of S and *μ *is the mean of *μ*_1 _and *μ*_2_.

The classification function is of the form:

G(x) = *λ*_1 _x_hue _+ *λ*_2 _x_saturation _+ *λ*_3 _x_intensity _- Constant     (6)

where G(x) is the classification score.

##### (b) Logistic Regression

Let x = {x_hue_, x_saturation_, x_intensity_} denote individual data structure present, X = {x_1_, x_2_, x_3_..., x_n_}, the training set and Y represent a column vector with class information of the test dataset. The probability of Y = 1 in a multiple logistic regression model [30] is given as

p = 1 / (1 + e^-*β*X^)     (7)

where *β *is the coefficients vector. The equation can be rewritten as

ln(p / (1-p)) = *β*X     (8)

Equation (8) represents the log of odds as a linear function of X. Since the values for log of odds is not available, a maximum likelihood function provides the solution.

Each dataset can be considered as a Bernoulli trial. That is, it is a binomial with the total number of trials equal to 1. Consequently for the i^th ^observation



Assuming all datasets are independent, the likelihood function is given by



The log of the likelihood function is given by



The parameter vector *β *are obtained by maximizing (11) using the efficient Newton-Raphson iterative technique. The classification function is of the form:

G(x) = *β*_0 _+ *β*_1 _x_hue _+ *β*_2 _x_saturation _+ *β*_3 _x_intensity _    (12)

where G(x) is the classification score.

The classification function was tested on the test set. If *z *= {z_hue_, z_saturation_, z_intensity_} is an individual data structure in the test set, it is classified as class 1 if G(*z*) > 0, otherwise, it is class 2. The classification scores were compared with actual scores and a classification table was constructed. If the percentage of class 1 nuclei and class 2 nuclei identified correctly is greater than 85, then the classification coefficients were used. If not, nuclei are randomly sampled again and the process was repeated.

Limits for nuclear area are added to the parameter set to eliminate possible artifacts in the image. The lower limit and upper limits of nuclear area were calculated from the dataset

Area _upper _= Area _Mean _+ 2SD     (13)

Area _lower _= Area _Mean _- 2SD     (14)

where SD is the standard deviation of the nuclear area measures.

### 5. Image analysis

**Figure 3:**
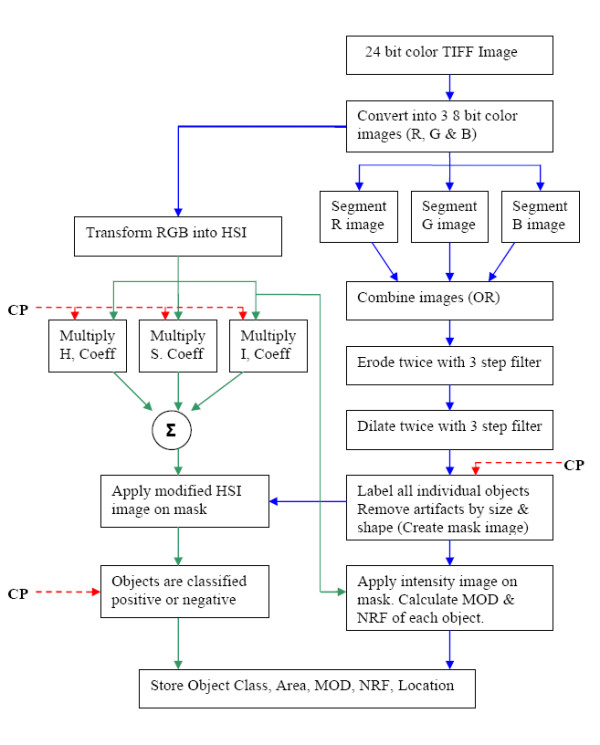
Block diagram of the image analysis program. CP – Classification Parameters, MOD – Mean Optical Density, NRF – Nuclear Roundness Factor, OR – Logical OR operation.

A block diagram of the image analysis program is shown in Figure 3. Red, green and blue color information was extracted from the original uncompressed 24-bit color image and stored as 8-bit grayscale images. Discriminant analysis of grayscale histograms was used to determine optimal thresholds for automated segmentation of red, green and blue images [31]. The adaptive threshold was applied using an 80 × 80 pixel window. This window size was chosen because it is about four times the size of a typical nucleus (nuclear diameter ~20 pixels). The three segmented images were combined by a logical OR operation. The combined image was eroded and dilated twice using a 3 step erosion filter (3 × 3 cross, 1 × 3 horizontal and 3 × 1 vertical kernels). Erosion was used to shrink the detected nuclear boundaries and dilation was used to fill the nuclear areas. Artifacts were removed based on size and shape. The nuclear regions were then labeled in raster fashion to create a nuclear mask. Regions not labeled are regarded as background.

**Figure 4:**
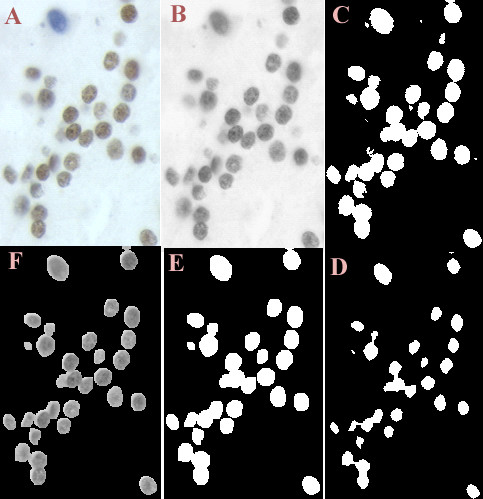
(clockwise). A – Part of the original image, B – Intensity image, C – Image after addition of R, G and B segmented images, D – Image after erosion (twice), E – Image after dilation (twice), F – Nuclear mask with intensity information.

Use of red, green and blue images to separate immunopositive from immunonegative nuclei is problematic because the color of stain is mixed with the intensity of stain. An HSI color model was used because it decouples intensity information from color information [32]. The hue, saturation and intensity component images were multiplied by their corresponding discriminant coefficients from the parameter file and combined to form a single image. A nuclear mask was applied to the image and the resulting nuclear areas were classified as immunopositive or immunonegative depending on their classification score. Figure 4 shows part of an image at different stages of image processing. The precise number of nuclei measured may be inaccurate due to the presence of nuclear overlap or clusters of nuclei. Addition of an upper limit for nuclear area measurement creates a reproducible error. Nuclear shape limits were also used to separate epithelial nuclei from artifacts, endothelial and stromal nuclei and inflammatory cells.

The nuclear mask was applied on the intensity image to obtain the intensity mask image. MOD of each nuclear area present in the image is calculated as:



where N is the total number of pixels in a nuclear mask, I_i _is the intensity level of the pixel i, and I_o _is the intensity level of the background measured in each field of view. NRF is the ratio of the radius of the circle the perimeter of which is equivalent to the measured perimeter to the radius of the circle of which is equivalent to the measured area. The NRF of each nuclear object is given by:



where A is the measured nuclear area and perimeter P is calculated using a chaining approximation, using weights (1, 4, 6, 4, 1). More information on MOD, area and perimeter calculations can be found in earlier publications [13,23].

## Results

The classification coefficients were computed using linear discriminant analysis and by logistic regression. There was no statistical difference in the results obtained by the two methods (Student's t test, P = 0.3896). The classification table is shown in Table 1. The percent positive nuclei and percent nuclear area were similar by race in both benign prostate hyperplasia and prostate cancer (Table 2). In prostate cancer epithelial nuclei, African Americans exhibited 38% higher levels of AR immunostaining than Caucasian Americans (two sided Student's t-tests; P < 0.05). Intensity of AR immunostaining was similar between races in benign prostate.

**Table 1:** Classification Table. AR – Androgen Receptor.

	**Predicted**	
	
**Actual**	Number of AR Positive nuclei	Number of AR Negative nuclei
Number of AR positive nuclei (106)	104 (98.11%)	2 (1.89%)
Number of AR negative nuclei (136)	2 (1.47%)	134 (98.53%)

**Table 2:** Androgen receptor expression in benign prostate and prostate cancer tissue specimens from 20 African Americans and 20 Caucasian Americans. AA – African American, CA – Caucasian American, BP – Benign Prostate, CaP – Prostate Cancer, P value (two sample comparison).

	**MOD**		**Percent Positive Area**		**Percent Positive Nuclei**	
	
	**CaP**	**BP**	**CaP**	**BP**	**CaP**	**BP**
**AA**	0.34 ± 0.09	0.32 ± 0.06	0.37 ± 0.20	0.45 ± 0.22	0.25 ± 0.15	0.28 ± 0.18
**CA**	0.25 ± 0.06	0.30 ± 0.08	0.38 ± 0.25	0.35 ± 0.18	0.26 ± 0.12	0.25 ± 0.11
	P < 0.05	P > 0.05	P > 0.05	P > 0.05	P > 0.05	P > 0.05

## Discussion

The differences measured in the intensity of AR expression in prostate cancer were consistent with previous studies. Gaston et al. [33] reported higher AR expression in benign prostate hyperplasia and prostate cancer in African Americans in a comparison of 25 African Americans and 25 Caucasian Americans. Higher levels of AR expression were also found in malignant glands from a homogenous population of native African men when compared to similar tissues from Anglo-Saxon Caucasian men [34]. Small sampling size and non-matched African American and Caucasian American tissue specimens used in this pilot study may account for the similarity of AR expression in benign prostate hyperplasia.

The research need for accurate measurement of AR expression in archival specimens to dissect the role of AR in prostate pathology led to a 19 year quest to develop a robust and automated image analysis system. The system has been used to measure cellular proliferation, apoptosis and tumor morphology. The system was also used to develop a sampling strategy for prostatic tissue microarray [35]. However extensive user training and computational knowledge of the operating system was required to operate the system. The current version of the system is platform independent and can be easily shared across research groups. The software GUI is intuitive and requires minimal user training. The system was also found independent of the imaging system as long as images were acquired using the same protocol. The constraint in the current version of the software is the image size. Image size is required to be a multiple of the segmentation window (80 × 80 pixels) for optimal performance. The option to set background to predetermined grayscale value has made it possible to differentiate regions of protein proliferation in dual-labeled, paraffin-embedded prostate tissue [36]. New features added to the system can be made available to all users by web-based transfer.

Two methods to compute the classification coefficients are presented. The choice between the two methods depends on the training set data. If the data is found (or assumed) to come from multivariate normal distribution, linear discriminant analysis is computationally more efficient. If the multivariate assumption is violated, logistic regression should be used [37].

The program performs local segmentation to detect nuclei and then uses color information to classify nuclei. Linear discriminant analysis is employed at the outset to generate classification coefficients. This priori information is required due to non-standardized immunostaining and image collection methods across medical institutions and research laboratories. The addition of classification parameters to the program customizes the software for the specimen under study. Nuclear shape information measured from each nuclear object can be combined with other morphologic descriptors to predict clinical, diagnostic and prognostic outcomes in prostate cancer [13]. This new automated method analyzes an image and provides reproducible measurements in less than 10 seconds. The availability of a free, automated system creates new opportunities for testing, evaluation and use of this image analysis system by many research groups who study nuclear protein expression.

## Authors' contributions

All authors have contributed equally in the creation of this manuscript.

## References

[B1] Jemal A, Murray T, Ward E, Samuels A, Tiwari RC, Ghafoor A, Feuer EJ, Thun MJ (2005). Cancer Statistics. A Cancer J Clin.

[B2] Scott WW, Menon M, Walsh PC (1980). Hormonal therapy of prostatic cancer. Cancer.

[B3] Tsai MJ, O'Malley BW (1994). Molecular Mechanisms of Action of Steroid/Thyroid Receptor Superfamily Members. An Rev Biochem.

[B4] Brolin J, Ekman P (1991). Microassays for androgen and progesterone receptor quantitation as compared with standard saturation analyses in human prostatic tissues. Urol Res.

[B5] Li R, Wheeler T, Dai H, Frolov A, Thompson T, Ayala G (2004). High level of androgen receptor is associated with aggressive clinicopathologic features and decreased biochemical recurrence-free survival in prostate: cancer patients treated with radical prostatectomy. Am J Surg Pathol.

[B6] Miyamoto KK, McSherry SA, Dent GA, Sar M, Wilson EM, French FS, Sharief Y, Mohler JL (1993). Immunohistochemistry of the androgen receptor in human benign and malignant prostate tissue. J Urol.

[B7] Sadi MV, Barrack ER (1993). Image analysis of androgen receptor immunostaining in metastatic prostate cancer. Heterogeneity as a predictor of response to hormonal therapy. Cancer.

[B8] Prins GS, Sklarew RJ, Pertschuk LP (1998). Image analysis of androgen receptor immunostaining in prostate cancer accurately predicts response to hormonal therapy. J Urol.

[B9] Tilley WD, Lim-Tio SS, Horsfall DJ, Aspinall JO, Marshall VR, Skinner JM (1994). Detection of discrete androgen receptor epitopes in prostate cancer by immunostaining: measurement by color video image analysis. Cancer Res.

[B10] Diamond DA, Berry SJ, Umbricht C, Jewett HJ, Coffey DS (1982). Computerized image analysis of nuclear shape as a prognostic factor for prostatic cancer. Prostate.

[B11] Mohler JL, Partin AW, Epstein JI, Becker RL, Mikel UV, Sesterhenn IA, Mostofi FK, Gleason DF, Sharief Y, Coffey DS (1992). Prediction of prognosis in untreated stage A2 prostatic carcinoma. Cancer.

[B12] Kim D, JD Charlton, Coggins JM, Mohler JL (1994). Semiautomated nuclear shape analysis of prostatic carcinoma and benign prostatic hyperplasia. Anal Quant Cytol Histol.

[B13] Veltri RW, Partin AW, Miller MC (2000). Quantitative nuclear grade (QNG): A new image analysis-based biomarker of clinically relevant nuclear structure alterations. J Cellular Biochemistry.

[B14] Mofidi R, Walsh R, Ridgway PF, Crotty T, McDermott EW, Keaveny TV, Duffy MJ, Hill AD, O'Higgins N (2003). Objective measurement of breast cancer oestrogen receptor status through digital image analysis. Eur J Surg Oncol.

[B15] Nabi G, Seth A, Dinda AK, Gupta NP (2004). Computer based receptogram approach: an objective way of assessing immunohistochemistry of androgen receptor staining and its correlation with hormonal response in metastatic carcinoma of prostate. J Clin Pathol.

[B16] Blatt RJ, Clark AN, Courtney J, Tully C, Tucker AL (2004). Automated quantitative analysis of angiogenesis in the rat aorta model using Image-Pro Plus 4.1. Comp Meth Prog Biomed.

[B17] Cell Analysis Systems Inc Cell analysis systems: Quantitative estrogen progesterone user's manual, Application Version 20, Catalog Number 201325-00, USA.

[B18] Acis^®^. http://www.chromavision.com/product/acis.htm.

[B19] Autocyte Pathology Workstation. http://www.tripathimaging.com/nonus_ac_quic_immuno.htm.

[B20] Schnorrenberg F, Pattichis CS, Kyriacou KC, Schizas CN (1997). Computer-aided detection of breast cancer nuclei. IEEE Trans Info Tech Biomed.

[B21] Xu YH, Sattler GL, Edwards H, Pitot HC (2000). Nuclear-labeling index analysis (NLIA), a software package used to perform accurate automation of cell nuclear-labeling index analysis on immunohistochemically stained rat liver samples. Comput Methods Programs Biomed.

[B22] Loukas CG, Wilson GD, Vojnovic B, Linney A (2003). An image analysis-based approach for automated counting of cancer cell nuclei in tissue sections. Cytometry.

[B23] Kim D, Gregory CW, Smith GJ, Mohler JL (1999). Immunohistochemical quantitation of androgen receptor expression using color video image analysis. Cytometry.

[B24] Kim D, Gregory CW, French FS, Smith GJ, Mohler JL (2002). Androgen receptor expression and cellular proliferation during transition from androgen-dependent to recurrent growth after castration in the CWR22 prostate cancer xenograft. Am J Pathol.

[B25] Gaston KE, Ford OH, Singh SS, Gregory CW, Weyel DE, Smith GJ, Mohler JL (2002). A novel method for the analysis of the androgen receptor. Curr Urol Rep.

[B26] Coggins JM (1992). Image and Graphics Library, Object-Oriented (IGLOO) Manual.

[B27] Java Web Start Reference Manual available. http://java.sun.com/products/javawebstart/developers.html.

[B28] UNC Prostate Cancer Research. http://www.med.unc.edu/lccc/iac/Protocols/downloads.htm.

[B29] Seber GAF (1984). Multivariate Observations.

[B30] Sharma S (1996). Applied Multivariate Techniques.

[B31] Otsu N (1979). A threshold selection method from gray-level histograms. IEEE Trans Systems Man Cybernetics.

[B32] Gonzalez RC, Woods RE (1992). Digital image processing. Reading, MA: Addison-Wesley.

[B33] Gaston KE, Kim D, Singh SS, Ford OH, Mohler JL (2003). Racial differences in androgen receptor protein expression in men with clinically localized prostate cancer. J Urol.

[B34] Olapade-Olaopa EO, Muronda CA, MacKay EH, Danso AP, Sandhu DP, Terry TR, Habib FK (2004). Androgen receptor protein expression in prostatic tissues in Black and Caucasian men. Prostate.

[B35] Singh SS, Qaqish B, Johnson JL, Ford OH, Foley JF, Maygarden SJ, Mohler JL (2004). Sampling Strategy for Prostate Tissue Microarray for Ki-67 and Androgen Receptor Biomarkers. Anal Quant Cytol Histol.

[B36] Ford OH, Singh SS, Miller SC, Smitherman AB, Lasater M, Mohler JL Dual labeling of bromodeoxyuridine and other antigens of interest in archival formalin-fixed, paraffin-embedded tissue for computer assisted image analysis.

[B37] Krzanwski WJ (1975). Discrimination and classification of both binary and continuous variables. J Am Stat Assoc.

